# Supplementary material for: Rational design of complex phenotype via network models
Source: PLoS Comput Biol. 2021 Jul 29;17(7):e1009189. doi: 10.1371/journal.pcbi.1009189 (PMC8354484; doi:10.1371/journal.pcbi.1009189)
Supplement: Attachment — Submitted filename: Mischaikow.pdf [file pcbi.1009189.s001.pdf]

This article presents an analysis of different hypothesized genetic networks that could underlie bistability and hysteresis. The authors exploit the Dynamic Signatures Generated by Regulatory Networks (DSGRN) methodology that they have developed in recent years. I am familiar with many aspects of these techniques from past interactions with the authors, but I am not expert in them. The technique is applied in the current paper to rank robustness of networks that can generate hysteresis. The key notion is that using geometrical and combinatorial techniques one can get a good idea concerning dynamics and robustness of a complex network (and the differential equations that might represent them), without integrating the equations, but simply by analyzing properties of graphs representing the network.

The basic approach is related to earlier work. Previous papers going back to Jacob and Monod, and including Stuart Kauffman, Rene Thomas, Rod Edwards, myself and many others, have proposed simplified models for genetic networks that could support bistability and hysteresis, and proposed graphical and algebraic techniques for analyzing dynamics. Although some of these earlier papers do address questions of robust dynamics in genetic regulatory networks, the current approach analyzes robustness of hysteretic dynamics in a new way by considering the way in which dynamics depends on parameters as an input is varied using the novel context of DSGRN to conclude that certain interaction networks are more robust than others. This type of conclusion goes beyond what would be possible using earlier methods and as such, could be valuable to the biologic community – especially to those who might wish to design hysteretic networks, but who do not have the mathematical expertise to carry out the needed computations.

To me, a central idea resides in the state transition graph, which represents flows between neighboring regions of state space. The description of the state transition graph on lines 90-95 is not clear to me. My understanding of the term is based on previous knowledge. As time proceeds variables cross thresholds thereby activating or inhibiting other variables. Representing volumes of phase space by points on a graph, the resulting directed graphs often capture essential features of the flow. A graph can be constructed simply based on a knowledge of the activation and inhibitory interactions in the network and the particular set of parameters and input.

There are some points that require clarification.

Symmetry. Are there really 19,683 distinct 3 node networks? Aren't some the same due to symmetry? (Glass, 1975; Edwards and Glass, 2000). I believe that one of the relevant state transition graphs for networks 1,2,3,4 in this paper corresponds to structure XII in my 1975 paper and one possible realization for network 2 in the bistable parameter range in the current paper is equation 22 in Glass (1975). According to my understanding the flows in networks 1,2,3,4 for a given set of parameters could all be the same under some symmetry transformation of the phase space vector fields. Presenting the state transition graph for a set of parameters in the bistable region for each would help clarify. I do not understand why you say these have different dynamical properties, so perhaps there is something that I am not getting right here. Likewise, I do

not understand why networks 11 and 12 here are considered different. Is it just because you are reading out from element 2?

Multiple inputs to a given node. In work by myself and others, there was the possibility that two activating inputs to a node could either be modeled by AND or OR logic (multiplication or addition of Hill functions). For example, Glass and Pasternack, (1978) use NAND functions and their continuous analogues in construction of a stable 4D oscillator. The additive functions in Eq. 1 here seem to preclude that. Likewise, if a node has both a positive and a negative input, it should be possible to have one of those always dictate the outcome. You should make clearer whether such possibilities exist in your formulation and whether the robustness of the dynamics would be affected by the specific ways multiple inputs to one variable interact.

Robustness. Earlier papers dealing with robustness often discuss the concept with regard to oscillations or sequential transitions. Based on the state transition diagrams one can eliminate what are called “critical race conditions” in circuit design. The robustness of bistability here seems a bit different, but I think there are common elements. Could you clarify how your concept of robustness here compares to earlier concepts as given for example in Perkins et al. (2010), Schmal et al. (2010)?

Practical applications. Naturally occurring systems can show bistability and hysteresis. Likewise there have been new synthetic bistable switches following the first from Collins group, for example Kim and Winfree (2006). Do natural or man-made bistable systems follow the structural designs that are determined here to be most robust? If so, this would strengthen the current analysis.

It would have helped me enormously in reading this paper and in writing this review, if the authors had given an example in which they made their computational techniques clear for one of the structures 1-4, and shown how their analysis supersedes and improves on earlier analyses that omitted key features included here, e.g., the possibility that a variable will have different thresholds for activation (or repression) of different variables. Working through an example of two networks, one more robust than the other would improve insight into the robustness of the networks. Can you predict which network will be robust by the interaction graph, without going through lengthy computations?

Overall, the paper does show a potentially useful way to attack robustness of bistability but the exposition for non-mathematicians would be improved by giving some clearer examples that could be appreciated with minimal jargon and technical notation. Relegating key methods to the end of the paper may conform to current guidelines but it also impedes comprehension. I would much prefer a de-mathematized methods section that gave a couple of examples in plainer language before the results.

Leon Glass

Glass, L. (1975). Combinatorial and topological methods in nonlinear chemical kinetics. *The Journal of chemical physics*, *63*(4), 1325-1335.

Glass, L., & Pasternack, J. S. (1978). Stable oscillations in mathematical models of biological control systems. Journal of Mathematical Biology, 6(3), 207.

Edwards, R., & Glass, L. (2000). Combinatorial explosion in model gene networks. *Chaos: An Interdisciplinary Journal of Nonlinear Science*, *10*(3), 691-704.

Kim, J., White, K. S., & Winfree, E. (2006). Construction of an in vitro bistable circuit from synthetic transcriptional switches. Molecular systems biology, 2(1), 68.

Perkins, T. J., Wilds, R., & Glass, L. (2010). Robust dynamics in minimal hybrid models of genetic networks. *Philosophical Transactions of the Royal Society A: Mathematical, Physical and Engineering Sciences*, *368*(1930), 4961-4975.

Schmal, C., Peixoto, T. P., & Drossel, B. (2010). Boolean networks with robust and reliable trajectories. *New Journal of Physics*, *12*(11), 113054.
