# Supplementary material for: Rational design of complex phenotype via network models
Source: PLoS Comput Biol. 2021 Jul 29;17(7):e1009189. doi: 10.1371/journal.pcbi.1009189 (PMC8354484; doi:10.1371/journal.pcbi.1009189)
Supplement: Attachment — Submitted filename: Response_Letter.pdf [file pcbi.1009189.s002.pdf]

Response to reviwer's report on the paper

Rational design of complex phenotype via network models

by Marcio Gameiro, Tomáš Gedeon, Shane Kepley, and Konstantin Mischaikow

(submitted to *PLOS Computational Biology*)

We would like to thank the reviewer for the detailed analysis of our manuscript and for the comments and suggestions regarding the results and organization of the paper. In response to the reviewer's suggestions we have significantly reorganized and extended the paper to try to make the technical parts of DSGRN more clear to the reader.

However, we decided to leave a more complete description of DSGRN for a future survey paper on DSGRN because that would take more space is reasonable to use in this paper. The collection of cited material fully describes DSGRN, but we agree that a more accessible survay paper would be a good idea.

Additionally we decided to maintain the main results of the paper at the beginning and leave the technical description of DSGRN for the Methods section. The main reason for this organization of the paper is that we intend for the results in this paper (the ranking of the networks) to be be used without a complete understanding of the DSGRN computation used to produce them. Hence we want to get first to the results and leave the more technical parts for later in the paper. We added a discussion of our reasons to present our results before explaining the details of DSGRN (lines 59-69 of the revised clean text or lines 59-69 in the blue text). We hope that our reorganization and additional material will make the paper more accessible and readable.

To facilitate the revision, with our resubmission we include a version of the revised manuscript where the text that was deleted or moved is presented in red and is crossed-out and new text appears in blue. We also include a clean version of the revised manuscript.

Below please find a detailed responses to all reviewer's suuggestions.

1. *Are there really 19,683 distinct 3 node networks? Aren't some the same due to symmetry? (Glass, 1975; Edwards and Glass, 2000). I believe that one of the relevant state transition graphs for networks 1,2,3,4 in this paper corresponds to structure XII in my 1975 paper and one possible*

realization for network 2 in the bistable parameter range in the current paper is equation 22 in Glass (1975). According to my understanding the flows in networks 1,2,3,4 for a given set of parameters could all be the same under some symmetry transformation of the phase space vector fields. Presenting the state transition graph for a set of parameters in the bistable region for each would help clarify. I do not understand why networks 11 and 12 here are considered different. Is it just because you are reading out from element 2?

Response: That is correct. The reason some networks are not the same due to symmetry is that we fix node 0 as the input and node 2 as the output node. We added a sentence to this effect (lines 88-90 of the revised clean text or lines 88-90 in the blue text)

2. *In work by myself and others, there was the possibility that two activating inputs to a node could either be modeled by AND or OR logic (multiplication or addition of Hill functions). For example, Glass and Pasternack, (1978) use NAND functions and their continuous analogues in construction of a stable 4D oscillator. The additive functions in Eq. 1 here seem to preclude that. Likewise, if a node has both a positive and a negative input, it should be possible to have one of those always dictate the outcome. You should make clearer whether such possibilities exist in your formulation and whether the robustness of the dynamics would be affected by the specific ways multiple inputs to one variable interact.*

   Response: Our assumptions do not exclude combination of inputs motivated by AND and OR logic. In fact, we assume that the inputs are combined in a form of "product of sums" where sum is motivated by the OR logic and product by the AND logic. To clarify this issue, we extended the discussion on the node inputs and outputs, including the choice of logic for the input edges (lines 383-406 of the revised clean text or lines 405-428 in the blue text).

3. *Earlier papers dealing with robustness often discuss the concept with regard to oscillations or sequential transitions. Based on the state transition diagrams one can eliminate what are called "critical race conditions" in circuit design. The robustness of bistability here seems a bit different, but I think there are common elements. Could you clarify how your concept of robustness here compares to earlier concepts as given for example in Perkins et al. (2010), Schmal et al. (2010)?*

   Response: Thank you for the suggestions of references for other concepts of robustness; we added them into the paper. In response we added Section 4.4 with an extensive discussion on the intuition into robustness and fragility. In this section we introduce two new Figures. In Figure 6 we illustrate our representation of hysteresis and in Figure 7 we illustrate on a state transition graph our concept of robust bistability.

4. *Naturally occurring systems can show bistability and hysteresis. Likewise*

there have been new synthetic bistable switches following the first from Collins group, for example Kim and Winfree (2006). Do natural or man-made bistable systems follow the structural designs that are determined here to be most robust? If so, this would strengthen the current analysis.

Response: We are currently working with two different groups of synthetic biologists to validate the theoretical predictions of this paper. Building of the requisite parts of the 3 node network requires significant effort and we plan to report on this work in a follow-up paper.

5. *It would have helped me enormously in reading this paper and in writing this review, if the authors had given an example in which they made their computational techniques clear for one of the structures 1-4, and shown how their analysis supersedes and improves on earlier analyses that omitted key features included here, e.g., the possibility that a variable will have different thresholds for activation (or repression) of different variables. Working through an example of two networks, one more robust than the other would improve insight into the robustness of the networks.*

   Response: We addressed this suggestion by introducing two new Figures 6 and 7 illustrating state transition graphs of two different networks and wrote a completely new section 4.4 on robustness and fragility. We also significantly expanded section where we compare DSGRN and Boolean models (lines 330-351 of the revised clean text or lines 342-363 in the blue text). Finally, we expanded our explanation of the parameter graph (lines 420-435 of the revised clean text or lines 454-469 in the blue text) in Methods section.

6. *Can you predict which network will be robust by the interaction graph, without going through lengthy computations?*

   Response: This is a very difficult task and we would love to be able to predict robustness based on a limited intutively accessible analysis of either interaction graph or a state transition graph. However, after analysing many networks we are convinced that it is the parameters encoded in the parameter graph that determine state transition graph, that ultimately decide the dynamical behavior of the system. Structure of the network determines parameter graph, but not in an obvious way. We believe that there is limited number of generalizations beyond the well understood "rules": positive loops beget multistablity and negative loops are related for existence of oscillations. In fact, DSGRN was developed as an computational tool to enumerate possible dynamics for each interaction graph to supplement the above rules as well as motif theory, which often has caveats and exceptions.

   However, we added Figure 7 to illustrate idea of robustness on the level of state transition graph.

7. *The exposition for non-mathematicians would be improved by giving some clearer examples that could be appreciated with minimal jargon and techni-*

cal notation. Relegating key methods to the end of the paper may conform to current guidelines but it also impedes comprehension. I would much prefer a de-mathematized methods section that gave a couple of examples in plainer language before the results.

Response: Thank you for this suggestions. We have had vigorous debate on the presentation of the paper because of this suggestions and this is the main reason why it took us a long time to resubmit the paper. At the end we decided to significantly expand the Methods section but to maintain the main results of the paper at the beginning. The main reason for this is that we intend for the main results in this paper (the ranking of the networks) to be be used without a complete understanding of the details of DSGRN computations.
