# Supplementary material for: Rational design of complex phenotype via network models
Source: PLoS Comput Biol. 2021 Jul 29;17(7):e1009189. doi: 10.1371/journal.pcbi.1009189 (PMC8354484; doi:10.1371/journal.pcbi.1009189)
Supplement: Attachment — Submitted filename: Response_Letter_New.pdf [file pcbi.1009189.s003.pdf]

Response to reviwer's report on the paper

Rational design of complex phenotype via network models

by Marcio Gameiro, Tomáš Gedeon, Shane Kepley, and Konstantin Mischaikow

(submitted to *PLOS Computational Biology*)

We would like to thank the reviewer for the comments and suggestions regarding the results and organization of the paper. We have modified the paper to address the reviewer's suggestions.

In addition to correcting the small typos we provide a response below to each one the additional suggestions by the reviewer.

1. *Symmetry. Networks 1-4 all have the same state transition diagrams (an example of which is now given in Fig. 6) and all have the same hysteresis scores in Fig. 8. From a computational perspective, and a dynamics perspective these seem to be the same network as classified by the symmetry under the 3-cube as demonstrated initially in Glass (1975), Edwards and Glass (2000). The added sentence in response to my earlier comment should be expanded to clarify so that a clever reader who recognizes that the symmetry may be relevant would realize that some aspects of symmetry in these networks have already been investigated.*

   Response: We expanded the discussion on the effects of symmetry on the hysteresis score.

2. *Multiple inputs to a node. The state transition diagrams will be very different depending on how multiple inputs combine (e.g. for two positive inputs as an AND or an OR function). Are the hysteresis scores independent of the type of interaction? This would seem highly unlikely to me, but a clear statement about the dependence of the hysteresis score on the implied function for multiple inputs would be useful.*

   Response: For this paper we adopted the convention that activating edges are added together and then are multiplied by repressing edges. Modifying this type of interaction would likely modify the hysteresis score. However the software is capable to perform the computations with any other type of interaction selected by the user.

3. *Dynamics of natural systems with hysteresis. As I pointed out in my first review, there are several earlier papers identifying natural and man-made networks with multiple steady states and hysteresis. It is great that the authors are collaborating with synthetic biologists to generate robust networks based on their insights, but I still believe that the authors should at least mention some of the earlier papers that have demonstrated or analyzed multiple steady states and indicate how they fit into the current scheme. For example, the current work would be strengthened if the circuits for plant development from Elena Alvarez-Buylla's group conformed to their robust networks. If the networks did not conform, the authors could predict that additional control elements should be present (a prediction almost certain to be correct!).*

   Response: We added a paragraph into the discussion acknowledging these papers and the fact that this is a related idea.

4. *Robustness. The authors now mention two earlier papers that deal with robustness, but do not give a clear indication of how the current formulation relates to the earlier ones. Although the current paper deals with hysteresis, presumably it could also deal with limit cycle dynamics. If so, would the earlier concept of stable attracting paths in the state transition diagram leading to robust dynamics match the formulation of the current work?*

   Response: The notion of robustness we discuss in the paper is robustness with respect to changes in parameters (changes in the parameter graph). We added and modified a few sentences in the paper to make it more clear.

   The DSGRN approach can and has been used to identify potential limit cycles. The question concerning robust dynamics in this context is interesting and largely unexplored at this point in time.
